# Research progress on tumor extracellular matrix stiffness and immunosuppression

**DOI:** 10.3389/fimmu.2026.1852616

**Published:** 2026-05-29

**Authors:** Fei Wu, Po Zhang, Weichi Wu, Tian Hou, Xiaobing Jiang, Tao Huang

**Affiliations:** 1 Department of Neurosurgery, Union Hospital, Tongji Medical College, Huazhong University of Science and Technology, Wuhan, China; 2 Division of Hematology/Oncology, University of Pittsburgh Medical Center (UPMC) Hillman Cancer Center, Pittsburgh, PA, United States; 3 Clinical Medicine, School of Medicine, Wuhan University of Science and Technology, Wuhan, China

**Keywords:** combination therapy, extracellular matrix stiffness, immunosuppression, mechanotransduction, tumor microenvironment

## Abstract

Tumor matrix stiffness, a pivotal physical attribute of the tumor microenvironment, has evolved from a passive physical barrier to an active immunoregulatory platform, profoundly impacting the initiation and effector phases of anti-tumor immune responses. This review systematically elaborates on the dual mechanisms that drive immunosuppression. Directly, stiffness attenuates T-cell and natural killer (NK) cell functions by activating pathways such as Yes-associated protein (YAP)/Transcriptional co-activator with PDZ-binding motif (TAZ) and Piezo-type mechanosensitive ion channel component 1 (Piezo1). It also drives the polarization of macrophages and dendritic cells towards immunosuppressive phenotypes. Indirectly, stiffness fosters an immune escape ecosystem by persistently activating cancer-associated fibroblasts, inducing tumor cell epithelial-mesenchymal transition, and upregulating immune checkpoints. Consequently, strategies such as enzymatic degradation, targeting mechanotransduction pathways, employing anti-fibrotic drugs, and developing intelligent combination therapies have emerged, aiming to soften tumors and reverse immunosuppression. Clinical studies confirm that high expression of the mechanosignaling hub Yes-associated protein 1 (YAP1) is associated with resistance to immunotherapy. In the future, integrating mechanobiology, immunometabolism, and smart materials to develop precise multimodal combination strategies holds promise for reversing the “cold tumor” microenvironment and opening new avenues to overcome immunotherapy resistance in solid tumors.

## 1 Introduction

For a long time, the focus on tumors has centered on genetic mutations and biochemical signaling pathways. Within this traditional framework, research on the tumor microenvironment (TME) has largely concentrated on its abundant signaling factors. However, the TME is not merely a biochemical reservoir; it is also a physical tissue with significant mechanical heterogeneity. Numerous studies indicate that variations in these physical properties—such as stiffness, elasticity, and fluid pressure—can be sensed by cells through specific mechanosensing mechanisms, including the integrin-focal adhesion kinase (FAK) and Yes-associated protein (YAP)/transcriptional co-activator with PDZ-binding motif (TAZ) pathways. These physical cues are then converted into biological signals, a process known as “mechanotransduction” (1–3). Furthermore, such mechanical signals play a widespread role within the immune system. Their effects are ultimately manifested in the critical clinical phenotype distinction between “immune-cold” and “immune-hot” tumors, thereby bridging the physical world and immunobiology (4).

The TME constitutes a complex ecosystem, with significant variations in its cellular and molecular composition across different cancer types. Studies have shown that even commonly used preclinical mouse models develop a TME characterized by unique immune cell compositions, CAF phenotypes, collagen content, and tissue stiffness (5), underscoring the complexity of the TME. Among these variables, the stiffness of the tumor extracellular matrix (ECM) stands out as one of the most prominently characterized and extensively studied physical parameters (6, 7). An increasing body of research reveals the underlying mechanisms by which matrix stiffness drives immunosuppression, opening a new dimension for understanding tumor immune evasion. A stiff matrix microenvironment is not merely a passive physical barrier but an active immunoregulatory platform (8). It directly or indirectly modulates immune cells, stromal cells, and tumor cells, thereby shaping an immunosuppressive ecosystem conducive to tumor growth. This type of mechanical barrier, alongside other biochemical immunosuppressive mechanisms, impedes immune cell infiltration and function, thereby severely limiting the successful application of immunotherapies. Consequently, a deeper understanding of “TME mechano-immunology”—the interplay between mechanical forces and immune cell behavior—is crucial for developing effective therapies for solid tumors (9). This review aims to systematically elucidate the mechanisms by which ECM stiffness drives immunosuppression through direct and indirect pathways, and to explore emerging strategies for enhancing immunotherapy efficacy by targeting this mechanical dimension.

## 2 Matrix stiffness-driven direct immunosuppression

In the TME, the abnormally deposited and remodeled ECM, particularly the density and stiffness of its collagen components, serves as a key physical factor that directly suppresses immune cell function (10). A rigid matrix orchestrates anti-tumor immune responses through multiple direct pathways: it regulates T cell differentiation and function, induces T cell exhaustion and promotes Treg polarization, while hindering T cell infiltration into the tumor parenchyma; it reshapes myeloid cell phenotypes, driving macrophages toward M2 polarization, suppressing dendritic cell (DC) maturation and antigen-presenting capacity, and promoting myeloid-derived suppressor cell (MDSC) expansion; it also significantly impairs natural killer (NK) cell cytotoxic function. These mechanisms collectively constitute the core network of stiffness-driven direct immunosuppression.

**Figure 1:**
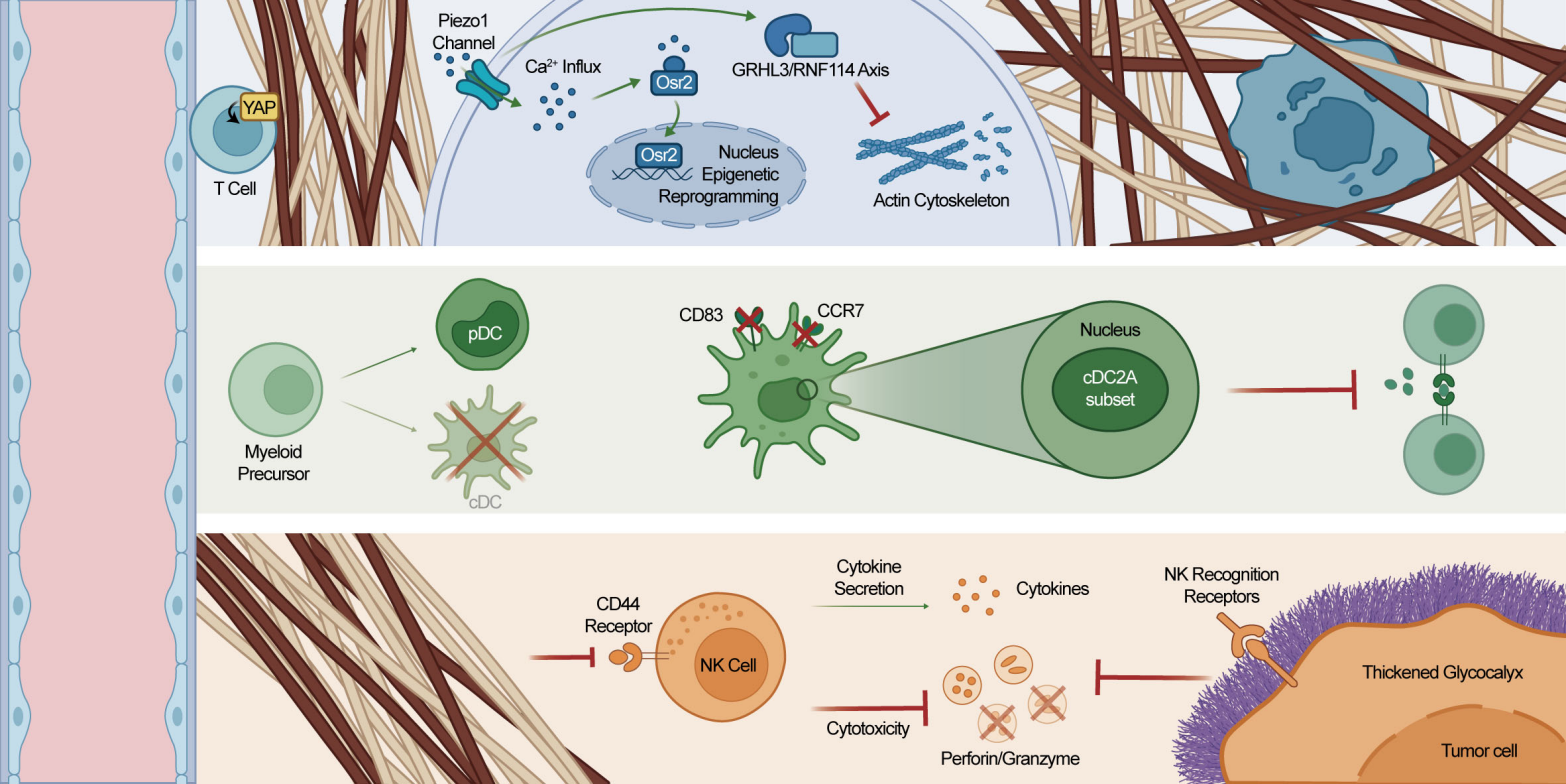
Hard tumor matrix drives immune escape by suppressing effector immune cell function. High-stiffness ECM inhibits T cell activation, proliferation, and cytotoxic function through mechanotransduction pathways such as YAP/TAZ and Piezo1, suppresses dendritic cell maturation and antigen presentation, and impairs natural killer cell cytotoxicity.

**Figure 2:**
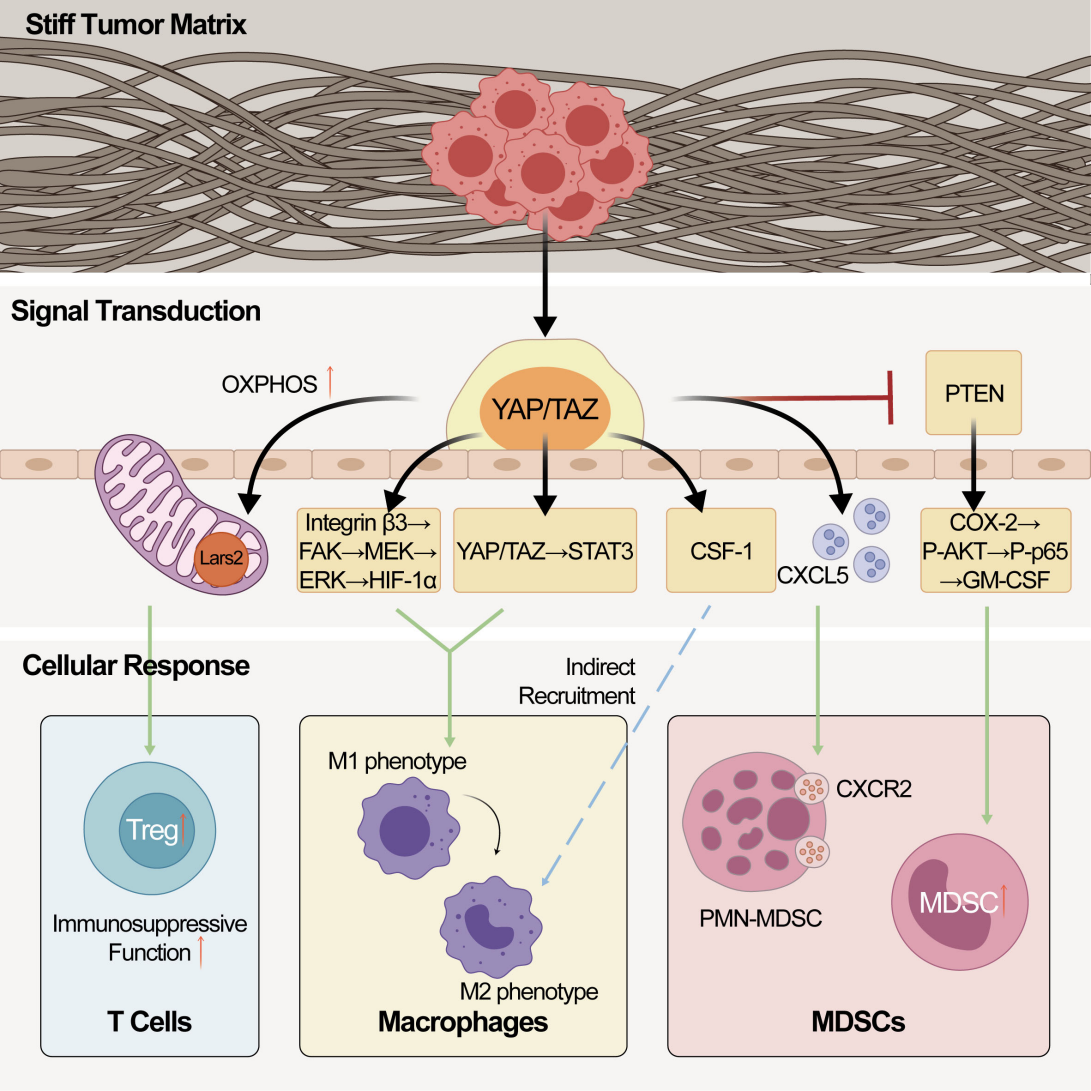
Hard tumor matrix enhances immunosuppression by promoting immunosuppressive cell populations. High-stiffness ECM induces regulatory T cell polarization, M2 macrophage conversion, and myeloid-derived suppressor cell expansion through mechanotransduction pathways such as YAP/TAZ and Piezo1, enhancing their immunosuppressive function.

Hard tumor matrix drives immunosuppression through a dual direct mechanism. On one hand, it directly suppresses the function of effector immune cells (such as T cells and NK cells) by activating mechanotransduction pathways like YAP/TAZ and Piezo-type mechanosensitive ion channel component 1 (Piezo1), inducing their exhaustion, dysfunction, or apoptosis. On the other hand, through the same mechanotransduction pathways, it actively promotes the expansion, polarization, and functional enhancement of immunosuppressive cell populations (such as regulatory T cells, M2 macrophages, and myeloid-derived suppressor cells). This dual action, suppressing anti-tumor immunity while promoting pro-tumor immunity, collectively constructs a microenvironment conducive to tumor immune escape. (**Figure 1**, **Figure 2**).

### 2.1 T cell dysfunction

In the TME, T cell dysfunction is a central link in immune evasion. ECM stiffness is a key factor directly leading to T cell dysfunction, and its mechanisms of action are primarily manifested in two aspects: functional impairment and physical infiltration barriers.

At the functional impairment level, a rigid ECM directly damages T cells through various signaling pathways. Studies using three-dimensional collagen models confirm that high matrix stiffness significantly inhibits T cell activation, proliferation, and cytokine production by enhancing YAP signaling (11). The activation of YAP/TAZ not only directly suppresses CD8^+^ and CD4^+^ T cells but also upregulates PD-L1 through interaction with STAT3, while simultaneously promoting the expansion of immunosuppressive regulatory T cells (Tregs), exacerbating immunosuppression across multiple layers (12). Additionally, the mechanosensitive ion channel Piezo1 impairs T cell cytotoxicity by upregulating the GRHL3/RNF114 axis, thereby disrupting the T cell actin cytoskeleton (13). The Piezo1/calcium/CREB signaling axis can also induce the expression of the transcription factor Osr2, which, by recruiting histone deacetylase HDAC3 for epigenetic reprogramming, directly drives CD8^+^ T cells toward terminal exhaustion (14) (**Figure 1**).

Beyond these well-defined biochemical signaling pathways, the physical properties of the matrix itself can also directly reshape T cell phenotypes. Research indicates that mechanical stress can broadly modulate the phenotypic spectrum of both resting and activated T cells (15). The viscoelasticity of the ECM can independently regulate T cell phenotype and function through the AP-1 signaling pathway (16). This finding suggests that the physical suppression mediated by the tumor matrix stems not only from its static stiffness but may also originate from its abnormal rheological properties.

The regulation of immune cells by matrix stiffness is bidirectional: while it suppresses effector T cells, it actively promotes and enhances the generation and function of Tregs. A stiff matrix reshapes cellular metabolic programs by guiding T cells to rely more on oxidative phosphorylation (OXPHOS) to meet their energy demands, actively steering them toward differentiation into immunosuppressive Tregs (17). Both 3D culture systems and TME studies confirm that increased matrix stiffness and viscoelasticity promote Treg differentiation, expansion, and migration(**Figure 2**), while simultaneously reinforcing their immunosuppressive function by regulating mitochondrial metabolism (18). Stiffness-activated YAP upregulates the expression of the mitochondrial protein Lars2, enhances OXPHOS, remodels Treg metabolic function, and thereby significantly boosts their immunosuppressive capacity (19).

At the physical infiltration level, ECM fibrosis driven by stiffness constitutes a direct barrier, impeding T cell migration and infiltration into the tumor parenchyma. *In vivo* studies using live human lung cancer slices visually demonstrate that dense and aligned matrix fibers confine T cells to perivascular areas, preventing their entry into tumor islands (20). This explains the phenomenon of T cell retention in the stromal regions of patient tumors. Quantitative studies further confirm a significant negative correlation between tumor stiffness and T cell migration efficiency (21).

### 2.2 Innate immune cell dysfunction

Matrix stiffness also profoundly reshapes the functional states of myeloid innate immune cells, such as macrophages and DCs. These cells not only respond to biochemical signals but are also highly sensitive to extracellular mechanical stimuli. Their recruitment, activation, metabolism, and effector functions are precisely regulated by physical signals (22). Studies using co-culture systems confirm that high stiffness can actively induce myeloid cell dysfunction, manifested by a general reduction in the co-stimulatory molecule CD86, alongside a consistent upregulation of pro-angiogenic factors (VEGFA), matrix metalloproteinases (MMPs), and the M2 marker CD206 (23).

#### 2.2.1 Macrophages

Tumor matrix stiffness precisely regulates tumor-associated macrophages (TAMs) phenotype and function through mechanotransduction, an emerging focus in tumor immunology (24). This regulation establishes a self-reinforcing vicious cycle that shapes the immunosuppressive microenvironment.

Matrix stiffness exerts precise and quantifiable control over macrophage polarization. Research indicates that macrophage polarization toward the anti-tumor M1 phenotype is only significantly activated when stiffness reaches a specific threshold (25). The stiffness range commonly found in solid tumors is typically below this threshold, which may account for the deficiency of M1 macrophages. Concurrently, stiffness can continuously and potently drive M2 phenotype polarization (**Figure 2**). The mechanisms involve multiple signaling pathways. On one hand, stiffness can directly induce M2 polarization and stimulate the secretion of the profibrotic factor lysyl oxidase-like 2 (LOXL2) through the integrin β3-FAK-MEK-ERK-HIF-1α axis, a mechanism confirmed in clinical samples of hepatocellular carcinoma (26). On the other hand, stiffness-activated YAP/TAZ can synergize with STAT3 to jointly drive the M2 polarization program (12). Furthermore, in breast cancer models, stiffness can indirectly recruit and enrich M2-like TAMs by driving high expression of colony-stimulating factor 1 (CSF-1) in tumor cells (27).

The mechanical remodeling of the matrix is not a unidirectional process. TAMs not only adapt to a stiff microenvironment but also actively participate in its construction, establishing a self-reinforcing vicious cycle. In breast cancer, activation of the atypical NF-κB signaling in TAMs drives the deposition of the proteoglycan HSPG2, directly leading to further ECM stiffening (28). In already stiffened matrices, TAMs guide cancer cell invasion and metastasis through precise mechanisms. At the tumor invasion front, the pro-metastatic function of TAMs is directly regulated by the YAP/TAZ mechanotransduction pathway. This pathway drives high expression of DAB2 in TAMs, which in turn mediates local ECM remodeling and directional migration of cancer cells by controlling integrin recycling, thereby facilitating invasive dissemination of cancer cells (29).

Beyond the overall stiffness of the matrix, the microstructure of collagen fibers itself serves as a critical physical signal for immune regulation. In pancreatic cancer, the extracellular protein βig-h3 guides macrophage polarization toward the pro-tumor M2 phenotype by binding to type I collagen and promoting the formation of thicker fibers; these M2 cells, educated by structured collagen, effectively suppress T cell immune responses when reinfused *in vivo (*30). This work demonstrates that the structure, not merely the abundance, of collagen can directly instruct the immunosuppressive function of macrophages.

Beyond its role in directly driving M2 polarization, the physical remodeling of the ECM itself can also influence macrophage dynamics by promoting their recruitment. Deformation fields generated within fibrillar collagen matrices provide far-reaching physical cues, attracting macrophages from hundreds of micrometers away (31). This mechanotactic migration is mediated by α2β1 integrin and stretch-activated channels, allowing macrophages to sense local displacement velocities of the ECM rather than relying solely on chemotactic gradients. Furthermore, dynamically reconstructed collagen fibers can transmit tensile signals that guide macrophage migration, with mechanosensitive ion channels and local calcium signaling playing key roles in this process (32). Thus, beyond being directly polarized by matrix stiffness, macrophages are also remotely recruited into stiffened tumor regions through ECM-mediated mechanical communication.

#### 2.2.2 DCs

Matrix stiffness is a fundamental environmental factor determining the differentiation and function of intratumoral myeloid cells, exerting influence throughout the entire process of DC development, differentiation, and immune activation. At the differentiation level, systematic research reveals a key pattern: on soft matrices, myeloid precursor cells primarily differentiate into conventional dendritic cells (cDCs) that initiate adaptive immunity; whereas on stiff matrices, they polarize towards immunosuppressive plasmacytoid dendritic cells (pDCs) and macrophage-like cells (23). Given that solid tumors typically exhibit high stiffness, and pDCs within the TME often display immunosuppressive functions (33), this finding strongly suggests that tumor matrix stiffening may actively shape a microenvironment conducive to the development of immunosuppressive pDCs but unfavorable for the differentiation of prototypical cDCs, thereby undermining the initiation of anti-tumor immunity at its source.

At the functional level, stiff matrices profoundly reprogram the functions of already differentiated DCs. *In vitro* studies confirm that DCs cultured on stiff matrices (~50 kPa) exhibit impaired antigen uptake, cell adhesion, and migration capabilities. More critically, their maturation process is also disrupted, manifesting as downregulated expression of CD83 and CCR7, leading to a severe decline in their ability to home to lymph nodes and activate T cells (34). This “functionally silenced” state is specifically validated in *in vivo* tumor models. In rhabdomyosarcoma, a high-stiffness microenvironment specifically suppresses the antigen cross-presentation capacity of the CD11b+ cDC2A subset through persistent activation of the mechanosensitive ion channel Piezo1, thereby weakening the anti-tumor response of CD8+ T cells(**Figure 1**). Genetic knockout of Piezo1 can reverse this suppression and significantly enhance tumor control (35).

#### 2.2.3 MDSCs

YAP/TAZ signaling, as a core component of mechanotransduction, serves as a cross-cancer hub driving myeloid immunosuppression, directly regulating the recruitment, expansion, and function of MDSCs (**Figure 2**). In pancreatic ductal adenocarcinoma, a typical fibrotic tumor, YAP within tumor cells directly promotes the differentiation and accumulation of MDSCs *in vivo* by driving the secretion of various cytokines and chemokines (36). A papillary renal cell carcinoma model further confirms that Yes-associated protein 1 (YAP1) activation is an initiating event for MDSC accumulation, and eliminating MDSCs blocks YAP1-driven tumorigenesis, establishing its causal role (37). The immunosuppressive mechanisms employed by YAP against MDSCs are diverse. In prostate cancer, the YAP-TEAD complex directly upregulates the chemokine CXCL5, which specifically recruits CXCR2+ PMN-MDSCs, driving tumor progression (38). In colorectal cancer (CRC), YAP1 actively induces MDSC expansion by inhibiting PTEN and activating the COX-2/P-AKT/P-p65/GM-CSF axis (39).

The mechanisms by which YAP/TAZ drive MDSC accumulation are diverse. Beyond recruiting MDSCs through the secretion of chemokines (such as CXCL5), research in CRC reveals that YAP1 actively induces the generation of MDSCs via a novel intracellular signaling axis. Specifically, YAP1 upregulates the levels of COX-2, P-AKT, and P-p65 by inhibiting PTEN expression, ultimately promoting the secretion of the key cytokine GM-CSF to induce MDSC expansion. In clinical CRC samples, YAP1, PTEN, and CD33+ MDSCs all serve as prognostic markers for patients, and each step of this mechanism has been functionally validated *in vitro (*40). This work unveils a new paradigm in which YAP shapes the immunosuppressive microenvironment by regulating the tumor cell’s intrinsic inflammatory signaling network.

#### 2.2.4 NK cell

NK cells in solid tumors are profoundly influenced by the ECM. The ECM not only constitutes a physical barrier limiting their migration but also directly modulates their functional state and indirectly interferes with their killing efficiency by altering the physical properties of target cells, forming a multi-layered immunosuppressive network.

A dense ECM presents the primary physical obstacle to NK cell infiltration. In highly fibrotic tumors such as pancreatic ductal adenocarcinoma, NK cells, although present and in an activated state, exhibit severely restricted invasion depth into the tumor parenchyma. Mechanistically, NK cells bind to ECM components via their surface CD44 receptor. This interaction, however, does not promote migration but instead anchors NK cells locally, actively hindering their advancement (41).

The ECM can directly reprogram the functional state of NK cells. A landmark study found that key ECM components, such as collagen I, III, and elastin, can switch NK cell function from direct cytotoxicity to cytokine/chemokine secretion. In skin graft and melanoma models, the presence of ECM directly shielded tumor cells from NK cell cytotoxicity, whereas pharmacological inhibition of tumor collagen deposition significantly restored NK cell killing capacity (42) (**Figure 1**).

The physical properties of the matrix can also indirectly lead to NK cell inactivation by remodeling the tumor cells themselves. In a breast cancer bone metastasis model, the mineralization of type I collagen—a core process causing bone matrix stiffening—was found to upregulate the sialylation modification of tumor cells, thereby thickening their surface glycocalyx. This physical barrier directly impaired NK cell recognition and attack of tumor cells. Thinning the glycocalyx using sialylation inhibitors significantly restored NK cell killing efficiency (43).

Mechanical regulation operates across multiple scales, from macro to micro. At the immune synapse level, a core mechanical process of NK cells themselves—the actomyosin retrograde flow (ARF)—precisely controls their cytotoxicity threshold. ARF drives the interaction between β-actin and the inhibitory phosphatase SHP-1, altering its conformation and establishing a mechanosensitive “brake” system for NK cell activation (44, 45).

However, the immunoregulatory effects of the matrix’s physical properties are not always inhibitory; they are context-dependent. For instance, when tumor cells grow on nano-grooved surfaces or are confined in elliptical patterns, their internal cytoskeletal tension increases, making them more sensitive to NK cell killing (46). This stands in stark contrast to the protective effect mediated by matrix mineralization, together revealing the complexity of how the physical microenvironment regulates NK cell function.

In summary, across different immune cell types, the mechanical signal of matrix stiffness is commonly sensed by integrins and Piezo1 and converges on shared downstream signaling hubs such as YAP/TAZ. This conserved mechanotransduction machinery enables effector T cells, NK cells, Tregs, macrophages, and MDSCs to respond to the same physical cue. The functional outcomes vary across cell types: effector populations are suppressed, whereas suppressive populations are reinforced. Nevertheless, the overall effect remains a synchronized shift of the entire immune landscape toward immunosuppression.

## 3 Matrix stiffness-driven indirect immunosuppression

Matrix stiffness drives multi-layered indirect immunosuppression by regulating stromal cells and tumor cells within the TME. In highly fibrotic tumors (such as hepatocellular carcinoma), activated hepatic stellate cells, as key stromal cells, collectively shape an immunosuppressive ecosystem by mediating stiffness signals, secreting ECM components, and releasing various factors (47). This process is often termed “fibro-inflammation,” a self-reinforcing cycle triggered by matrix stiffness, characterized by abnormal ECM deposition and stiffening as its physical foundation, and accompanied by persistent inflammation and immunosuppression (48). Within this framework, cancer-associated fibroblasts (CAFs), persistently activated by mechanical signals, indirectly weaken anti-tumor immunity by constructing physical barriers, remodeling the metabolic environment, and recruiting immunosuppressive immune cells. Concurrently, tumor cells undergo phenotypic transformation under stiffness stimulation, actively evading immune surveillance through mechanisms such as upregulating immune checkpoints, inducing epithelial-mesenchymal transition (EMT), and reducing immunogenicity. Together, they constitute the core effector units of stiffness-driven indirect immunosuppression. While other cellular components of the TME, such as endothelial cells or adipocytes, may also respond to mechanical cues, the current discussion focuses on CAFs and tumor cells as the best-characterized examples.

### 3.1 CAFs

CAFs are the most abundant stromal cells in the TME and serve as a central hub driving immunosuppression. They act as both responders to and primary producers of stiffness. Exhibiting significant heterogeneity, they can differentiate into various phenotypes such as myofibroblasts and inflammatory subtypes, performing multiple functions in tumor progression (49). In the context of tumor matrix stiffening, mechanical tension itself is a key physical signal driving and sustaining CAF activation. Research using three-dimensional microtissues confirms that high tension is sufficient to independently induce myofibroblast phenotypes, including α-SMA expression and YAP nuclear translocation. This process is reversible upon tension removal (50), providing a fundamental principle for understanding how a stiffened matrix continuously drives a pro-tumor stroma. The activation of CAFs and their role in mediating ECM protein accumulation and cross-linking are critical factors in regulating ECM remodeling. The resulting matrix stiffness profoundly influences the efficacy of immunotherapy (51).

Matrix stiffness activates CAFs through core mechanosignaling pathways such as YAP/TAZ and Piezo1, establishing self-reinforcing positive feedback loops. YAP/TAZ has been established as a key coordinator of stiffness-regulated fibroblast activation. In breast cancer, YAP drives cytoskeletal remodeling to stiffen the matrix, and the stiffened matrix, in turn, further activates YAP, creating a loop that stabilizes the activated state of CAFs (52, 53). Furthermore, the mechanosensitive ion channel Piezo1, in gastric and squamous cell carcinomas, drives CAF-mediated collagen deposition by activating YAP and TGFβ1 secretion, collectively forming a vicious cycle from matrix stiffness to mechanotransduction to CAF activation and further matrix stiffening (54, 55).

Activated CAFs drive immunosuppression through diverse mechanisms. Firstly, CAFs actively construct physical barriers: They actively stiffen the ECM by catalyzing collagen cross-linking via enzymes such as LOXL2 (56) and LH2/PLOD2 (57), and even modulate cross-link types. In pancreatic cancer models, this barrier can be disrupted by FAK inhibitors, thereby promoting CD8+ T cell infiltration (58). Secondly, CAFs shape a biochemical immunosuppressive milieu: They undergo metabolic reprogramming, forming a complementary metabolic symbiosis network with cancer cells (59); and by activating pathways like Rho/Hippo/YAP, they recruit and induce TAM polarization towards the M2 phenotype, establishing an axis of indirect immunosuppression (60).

In summary, CAFs synergistically impede anti-tumor immunity by constructing dense physical barriers and shaping an immunosuppressive environment. Targeting the mechanical signaling pathways that drive CAF activation is an effective strategy for reversing this immunosuppressive ecosystem.

### 3.2 Alterations in tumor cell behavior

Matrix stiffness alters tumor cell behavior through multiple mechanisms, driving the acquisition of immune escape capabilities. Matrix stiffness directly promotes immune evasion by inducing tumor cells to upregulate immune checkpoint molecules. Studies in lung adenocarcinoma show that a stiff matrix significantly increases PD-L1 protein expression through a mechanism dependent on cytoskeleton polymerization and stress fiber formation; this effect can be attenuated by inhibiting actin polymerization with cytochalasin D (61). In prostate cancer, high stiffness drives YAP nuclear translocation via the integrin β1/FAK/YAP axis. Once in the nucleus, YAP binds to TEAD2 and transcriptionally upregulates the deubiquitinase USP8, which stabilizes PD-L1 protein via K48-linked deubiquitination (62). Beyond PD-L1, stiffness also upregulates PD-L2 while suppressing the metalloreductase STEAP3 to inhibit ferroptosis, creating a dual barrier of death resistance and immunosuppression (63). Further research reveals that nuclear-localized FAK, as a hub for mechanical signals, directly drives CD8^+^ T cell exhaustion and Treg recruitment by transcriptionally regulating a chemokine/cytokine network, including CCL5. FAK inhibitors can reverse this immunosuppressive state and trigger CD8^+^ T cell-mediated tumor regression (64).

Concurrently, matrix stiffness indirectly promotes immune evasion by inducing and stabilizing the EMT program. Research reveals that stiffness and the core EMT transcription factor ZEB1 form a positive feedback loop: stiffness upregulates ZEB1 expression, which in turn promotes LOXL2 secretion to catalyze collagen cross-linking, further increasing matrix stiffness and locking tumor cells in a mesenchymal state (65). In melanoma, stiffness upregulates the chromatin remodeler SNF5 to activate STAT3 signaling, simultaneously enhancing both EMT and immune escape capabilities. Loss of SNF5 increases CD8^+^ T cell infiltration and reduces PD-L1-positive cells (66). Three-dimensional co-culture models confirm that stiffness and TAMs synergistically induce EMT, indicating that a stiffened matrix provides a critical physical permissive environment for TAMs to exert their pro-tumor and immunosuppressive functions (67).

Furthermore, matrix stiffness broadly affects anti-tumor immunity by modulating tumor cell immunogenicity and secretome. A stiff matrix activates the ROCK-myosin IIA-F-actin axis, promoting autophagic degradation of cGAS and thereby inhibiting the cGAS-STING innate immune pathway, which weakens tumor immunogenicity. Targeting this mechanical axis can restore cGAMP production and enhance immunotherapy efficacy (68). In pancreatic cancer, stiffness reprograms the proteome of tumor-derived extracellular vesicles, affecting the secretion of immune response-related molecules (69).

In summary, matrix stiffness establishes a self-reinforcing network among CAFs, tumor cells, and immune cells. Activated by YAP/TAZ and Piezo1, CAFs remodel the ECM and secrete immunosuppressive cytokines and chemokines, while tumor cells upregulate immune checkpoints and undergo EMT. These coordinated responses amplify the initial mechanical signal, transforming a localized physical cue into a persistent, globally immunosuppressive microenvironment.

## 4 Therapeutic strategies targeting matrix stiffness

The modulation of immune cells through mechanical means to enhance cancer immunotherapy is rapidly evolving into a burgeoning frontier. Current research in this field primarily revolves around the following directions: utilizing biomaterials with tunable properties to optimize T cell expansion and activation *ex vivo*; engineering T cells to secrete ECM-degrading enzymes to soften tumors and enhance infiltration; and even achieving spatiotemporally precise control of therapies like CAR-T *in vivo* through physical stimuli to mitigate off-target toxicity (70). The core principles of these strategies are detailed below.

### 4.1 Enzymatic intervention and ECM degradation

Dense ECM serves as a critical physical barrier that impedes immune cell infiltration and limits the efficacy of immunotherapy. Degrading the ECM using enzymatic approaches to soften the tumor matrix has emerged as a core strategy for improving responses to immunotherapy. Current enzymatic interventions primarily focus on two main directions: direct degradation of ECM components and inhibition of key enzymes responsible for matrix cross-linking.

#### 4.1.1 Direct degradation of ECM components

Direct cleavage of ECM components using enzymes can rapidly disrupt the physical barrier and enhance immune cell infiltration. The aforementioned research indicates that degrading the matrix with collagenase improves the ability of T cells to contact cancer cells (20), providing a crucial basis for using enzymatic intervention to enhance immunotherapy. To improve targeting, researchers have developed acid-responsive nanoformulations that can programmatically release hyaluronidase at the tumor site to degrade the ECM. In a pancreatic cancer model, this strategy significantly enhanced the deep infiltration and killing efficacy of NK92 cells (71), validating the effectiveness of direct enzymatic digestion of the ECM in improving immune cell delivery.

#### 4.1.2 Inhibiting key enzymes in matrix cross-linking

The lysyl oxidase (LOX) family is a core class of enzymes that mediate collagen cross-linking and drive matrix stiffening, making targeting LOX/LOXL2 an important approach for reversing fibrosis. Inhibiting LOX activity can reduce ECM deposition and lower tumor stiffness, thereby directly improving T cell migration capacity within tumors (21). Preclinical studies demonstrate that the potent LOX inhibitor PXS-5505 can significantly reduce matrix stiffness and fibrosis in pancreatic cancer models, and this softening effect improves tumor perfusion and enhances chemotherapy efficacy (72). Furthermore, inhibiting CAF-derived LOXL2 can disrupt ECM structure and suppress cell migration (56). These studies provide a mechanistic basis for combining LOX/LOXL2 inhibitors with immunotherapies.

#### 4.1.3 Multifunctional integration of enzymes in combination therapy

The application of enzymatic intervention has evolved beyond direct matrix degradation, and is now being integrated as a key functional component into more complex combination therapeutic systems. This aims to achieve precise temporal and spatial control over the treatment process. In a composite strategy against breast cancer, collagenase was incorporated into a multifunctional hydrogel. It serves to rapidly degrade the ECM and reduce interstitial pressure during the initial treatment phase, thereby significantly enhancing the intratumoral penetration of co-delivered photosensitizers, STING agonists, and oxygen. This lays the physical groundwork for the successful implementation of subsequent photodynamic and immunotherapy (73).

A more advanced design concept involves utilizing specific enzymes that are overexpressed locally in tumors as “biological switches” to control drug release. For instance, a smart delivery system (PCPP) can specifically recognize and respond to high concentrations of MMP-2 in the TME. Upon activation by MMP-2, this system releases low doses of cyclophosphamide to selectively deplete intratumoral Tregs. Concurrently, the system incorporates a light-controlled module capable of generating interleukin-2 *in situ* upon near-infrared light irradiation. This combination strategy has been proven effective in a liver cancer model for expanding and activating CD8^+^ T cells, achieving sequential modulation of the immune microenvironment (74). These advancements signify that enzymatic intervention is evolving into a core driver for synergistic multi-modal treatment regimens.

However, enzymatic intervention strategies face significant challenges and controversies on the path to clinical translation. Nonspecific or uncontrolled ECM degradation can be a double-edged sword. Foundational studies indicate that highly metastatic tumor cells inherently exhibit a greater propensity to degrade matrix and migrate, a phenotype that can be amplified by external signals (75). This suggests that indiscriminate ECM degradation may inadvertently mimic a pro-metastatic microenvironment, potentially increasing the risk of invasion. Furthermore, broad matrix degradation can compromise vascular integrity, leading to adverse effects such as hemorrhage (76). These potential risks underscore that future enzymatic interventions must strive for precision, controllability, and localization. The development of TME-responsive nanodelivery systems (e.g., pH-, enzyme-, or redox-responsive carriers) or engineered enzymes with higher substrate selectivity holds promise. These approaches aim to effectively soften the tumor barrier while maximizing the preservation of normal tissue homeostasis, thereby achieving a crucial balance between efficacy and safety.

### 4.2 Targeting mechanosignaling pathways

Matrix stiffness drives tumor fibrosis by activating specific signaling pathways and concurrently induces the expression of immunosuppressive molecules, constituting a key mechanism in the formation of an “immune-cold” tumor microenvironment. Consequently, targeting these mechanosignaling nodes has emerged as a crucial strategy for reversing immunosuppression and enhancing responses to immunotherapy. Current strategies primarily focus on three levels: targeting downstream key signaling proteins, intervening with upstream mechanical sensing receptors, and modulating the mechanical sensors within immune cells themselves.

#### 4.2.1 Targeting downstream key signal transduction proteins

In highly fibrotic pancreatic ductal adenocarcinoma (PDAC), heightened FAK activity is closely associated with tumor fibrosis and the paucity of CD8^+^ T cell infiltration. Treatment with the selective FAK inhibitor VS-4718 not only limits tumor progression and reduces fibrosis but also significantly improves the immune microenvironment (58). This confirms the feasibility of sensitizing immunotherapy by softening the matrix through FAK inhibition.

YAP/TAZ represents another crucial class of mechanosignal transduction proteins, and targeting this pathway shows great potential for reshaping the immune microenvironment. In 3D stiff matrix models, directly inhibiting the aberrantly YAP signaling activated by stiffness successfully restored T cell activation and immune response functions (11), indicating that targeting this pathway can directly overcome stiffness-induced T cell suppression. In breast cancer models, inhibiting YAP reversed the M2 polarization phenotype of TAMs and reduced PD-L1 expression by disrupting its interaction with STAT3, thereby promoting T cell attack and tumor suppression (12). This provides a theoretical basis for combining YAP/STAT3 inhibitors with existing immunotherapies.

Furthermore, targeting the RhoA/ROCK pathway, which regulates cytoskeletal contraction, can also effectively soften the matrix. For example, the multi-target tyrosine kinase inhibitor anlotinib has been shown to reduce CAF activation and ECM stiffness by inhibiting this pathway, creating favorable conditions for sensitizing subsequent treatments (77).

#### 4.2.2 Targeting upstream mechanosensing receptors: integrins

Beyond targeting downstream signaling molecules like FAK and YAP/TAZ, directly intervening at the source of mechanosensing—the cell surface integrin receptors—also constitutes an effective strategy for reversing immunosuppression. In CAFs, integrin α5 regulates ECM deposition, influencing the physical infiltration environment for T cells. Inhibiting α5 function can improve the matrix barrier, creating conditions conducive to T cell infiltration (78). In CRC, high expression of integrin αvβ6 on tumor cells activates TGF-β, mediating potent immunosuppression and resistance to PD-1 blockade. Antibody-mediated inhibition of αvβ6 can block this pathway, stimulate cytotoxic T cell responses, and generate strong synergistic effects when combined with anti-PD-1 therapy (79).

#### 4.2.3 Modulating the intrinsic mechanosensing of immune cells

Moving beyond modifying the TME, directly modulating the intrinsic mechanosensing apparatus of immune cells (such as T cells) represents a highly promising emerging direction. For instance, pre-treating T cells with antagonists of the mechanosensitive ion channel PIEZO1 can serve as a form of “mechanical vaccination,” significantly enhancing their tumor infiltration capacity and anti-tumor efficacy upon reinfusion (13). This immunomechanical modulation strategy provides a novel approach for enhancing the effectiveness of immunotherapies such as adoptive cell therapy.

In summary, strategies targeting mechanosignaling pathways (e.g., FAK, YAP/TAZ, integrins) and direct enzymatic degradation of the ECM are functionally complementary. The former focuses on correcting aberrant mechanotransduction signals, aiming to reverse stiffness-driven fibrosis and immunosuppression programs at their source. The latter primarily addresses the physical reshaping of the matrix barrier to improve infiltration. Theoretically, interventions centered on softening signals, which act on upstream signaling events leading to pathological phenotypes rather than directly dismantling tissue architecture, may offer a superior safety profile and controllability in the future. This suggests that combining highly selective signaling pathway inhibitors with spatiotemporally controlled local matrix remodeling strategies may represent the next-generation direction for balancing efficacy and safety.

### 4.3 Repurposing anti-fibrotic drugs

CAFs are central stromal cells driving tumor fibrosis, immunosuppression, and therapy resistance. Repurposing drugs already approved for non-neoplastic fibrotic diseases for cancer treatment has emerged as a highly promising strategy. The clinical anti-pulmonary fibrosis drug pirfenidone (PFD) exemplifies this approach, as it can inhibit CAFs, thereby restricting tumor proliferation, metastasis, immunosuppression, drug resistance, and reducing matrix stiffness (80). Simultaneously, losartan, a widely used antihypertensive medication, is being re-evaluated for its anti-fibrotic properties.

#### 4.3.1 Sensitizing immunotherapy

The core value of anti-fibrotic drugs lies in remodeling the immunosuppressive microenvironment, thereby creating synergy with immune checkpoint inhibitors (ICIs). In a lung cancer model with concurrent idiopathic pulmonary fibrosis, pirfenidone (PFD) enhanced the T-cell inflammatory signature of the tumor. Its combination with an anti-PD-L1 antibody significantly inhibited tumor growth, prolonged survival, and optimized intratumoral T-cell infiltration (81). In a breast cancer model, while losartan alone was ineffective, its combination with an anti-PD-L1 antibody significantly increased intratumoral CD8^+^ T-cell infiltration and granzyme B production. The therapeutic effect strictly depended on CD8^+^ T cells and effectively suppressed lung metastasis (82). In an ovarian cancer model, losartan systemically remodeled the microenvironment by improving vascular perfusion and inhibiting IGF-1 signaling, thereby enhancing the efficacy of combined chemotherapy and immunotherapy (83).

#### 4.3.2 Overcoming drug resistance and systemically remodeling the TME

Anti-fibrotic drugs are also employed to overcome complex resistance mechanisms and systemically remodel highly immunosuppressive “cold tumors” within multimodal combination therapies. In pancreatic cancer, CAFs induce drug resistance of tumor cells to the BET inhibitor JQ1 by upregulating BRD4 expression. Utilizing CAF-homing liposomes to deliver pirfenidone (PFD) can simultaneously reduce ECM production and downregulate BRD4, thereby reversing drug resistance both physically and functionally (84). In pancreatic cancer, monotherapy with gabapentin can induce resistance by upregulating TGF-β1. A nanodrug co-delivering gabapentin, PFD, and EGCG leverages PFD to antagonize TGF-β1 signaling, synergistically reversing resistance and reprogramming CAFs into an inflammatory phenotype. This strategy significantly promotes the infiltration of functional CD8^+^ T cells and substantially extends survival (85). In the highly fibrotic and immunosuppressive model of triple-negative breast cancer, losartan, serving as a matrix-depleting agent, is combined with doxorubicin liposomes and anti-PD-1 antibodies. By reducing the ECM and alleviating hypoxia, it promotes drug delivery and dendritic cell maturation while reprogramming M2 macrophages to an M1 phenotype. Ultimately, this significantly enhances T cell infiltration, generates potent synergy with immunotherapy, and inhibits tumor growth and metastasis (86).

### 4.3.3 Achieving precise and long-acting intervention

Nanotechnology and advanced formulations are pivotal for enhancing the efficacy of anti-fibrotic drugs and achieving precise intervention. Encapsulating PFD in polymeric micelles can effectively soften tumors at a dose 100 times lower than that of the free drug. Combining these micelles with ICIs significantly inhibits tumor growth, increases effector T cell infiltration, reduces MDSCs, and induces immune memory. This study also, for the first time, utilized ultrasound shear wave elastography to achieve non-invasive, dynamic monitoring of tumor stiffness (87). Targeting the characteristics of the pancreatic cancer microenvironment, researchers constructed an MMP-2-responsive peptide-liposome hybrid system (MRPL) for loading PFD. This system enables specific drug release at the tumor site, precisely downregulating ECM produced by pancreatic stellate cells, thereby significantly enhancing the intratumoral penetration and efficacy of the subsequent chemotherapy drug gemcitabine (88). PFD-loaded PCPP can degrade hyaluronic acid and collagen I, reducing matrix stress, thereby alleviating vascular compression, improving oxygenation, and enhancing the intratumoral accumulation of subsequent photosensitizers, ultimately greatly improving the efficacy of photodynamic therapy (89). To achieve long-term local softening, researchers developed injectable microspheres loaded with losartan potassium, co-encapsulated with cisplatin nanoparticles in a thermosensitive hydrogel. This system can durably inhibit tumor collagen I synthesis, significantly enhancing the intratumoral penetration and retention of chemotherapeutic drugs, resulting in potent local tumor control in a melanoma model (90).

#### 4.3.4 Enhancing traditional drug delivery

A fundamental application of anti-fibrotic drugs lies in improving the intratumoral delivery of traditional chemotherapeutic agents by softening the matrix. In a CRC model, PFD effectively softened the tumor matrix by inhibiting TGF-β1 signaling and reducing collagen deposition, thereby significantly enhancing the intratumoral penetration and accumulation of nano-formulated chemotherapy drugs and ultimately improving their anti-tumor efficacy (91). This strategy provides a universal approach to overcoming chemotherapy resistance caused by dense matrices.

The drug repurposing strategy for anti-fibrotic agents, by targeting CAFs and the ECM, offers a multifaceted solution for softening the tumor matrix, improving perfusion and oxygenation, and overcoming therapeutic barriers in solid tumors. This approach not only enhances the delivery of traditional chemotherapy but also synergizes with ICIs to reverse the “cold tumor” state. Furthermore, driven by technological innovations such as nanodelivery, it demonstrates potential for precise intervention and overcoming drug resistance. In the future, combining biomarker-guided precise application and integration with emerging therapies is expected to further enhance its clinical value.

### 4.4 Combination therapy

Tumor stiffness and immunosuppression together form a self-reinforcing vicious cycle. On one hand, the rigid ECM creates a physical barrier that directly hinders the infiltration of immune effector cells, such as T cells, into the tumor. On the other hand, the sustained mechanical signals transmitted by the matrix activate pathways like YAP and FAK, inducing both tumor cells and stromal cells to overexpress immunosuppressive molecules such as PD-L1 and TGF-β, collectively shaping the typical “immune-cold” tumor microenvironment (12). Within this cycle, monotherapies often prove ineffective: immunotherapy alone may fail because T cells cannot effectively infiltrate due to physical barriers or become functionally exhausted upon encountering strong immunosuppression. While softening the matrix alone can improve physical permeability, it cannot reverse established immune evasion mechanisms. Therefore, the core rationale for combination therapy lies in degrading the ECM or blocking mechanosignaling pathways to improve immune cell infiltration, while concurrently combining immunotherapy to restore their function. This strategy aims to fundamentally reverse an “immune-cold” tumor into an “immune-hot” state, thereby overcoming resistance to immunotherapy.

#### 4.4.1 Synergistic effects of matrix softening and ICI

This strategy directly addresses the dual bottlenecks of the physical barrier and immunosuppression, and has established several successful paradigms. Theoretical research indicates that targeting the matrix not only improves the physical infiltration of T cells but also directly enhances the functional restoration effect of ICIs by altering the mechanical microenvironment (92). At the practical level, multiple studies have confirmed the effectiveness of this strategy. In pancreatic cancer models, combining a therapy targeting the stiffness-related protein SLC20A1 with an anti-PD-L1/TGF-β bispecific antibody significantly promoted CD8+ T cell infiltration and enhanced their function (93). The combination of LOX inhibitors, which interfere with collagen cross-linking, with anti-PD-1 antibodies can markedly improve therapeutic outcomes by reducing matrix stiffness (21). Similarly, reducing ECM stiffness and reprogramming CAFs through photothermal therapy can create favorable conditions for PD-1 blockade, successfully reversing the immunosuppressive state in models such as cholangiocarcinoma (94). An even more innovative approach involves co-loading the chemotherapeutic drug oxaliplatin and anti-PD-L1 antibodies into an MMP-responsive smart hydrogel, enabling programmed local drug release at the tumor site. This strategy effectively inhibits tumor growth and elicits abscopal effects in melanoma models (95). Stabilizing intratumoral mast cells using the antihistamine ketotifen can inhibit CAF activity and reduce matrix stiffness, thereby enhancing the efficacy of chemotherapy combined with anti-PD-L1 therapy. The softening effect of this strategy has received preliminary validation in a sarcoma clinical trial (96). Directly targeting core molecules regulating the ECM in CAFs is another effective approach. In CRC, the high expression of integrin α5 in CAFs has been identified as a key node driving abnormal ECM deposition and hindering CD8^+^ T cell infiltration. Inhibiting α5β1 function reduces ECM deposition, increases T cell infiltration, and generates significant synergy with anti-PD-L1 therapy, improving treatment outcomes. Clinical data analysis further supports that high α5 expression is associated with T cell exclusion and poor immune prognosis (78).

#### 4.4.2 Synergy between matrix softening and adoptive cell therapy

For therapies reliant on substantial cellular infiltration, matrix softening strategies are integrated throughout the entire process of manufacturing, delivery, and action. Cultivating T cells in flexible 3D hydrogels that mimic the stiffness of soft tissues can promote their acquisition of a superior activation phenotype (97). Utilizing integrated biomaterial scaffolds that simulate the mechanical microenvironment of lymphoid organs not only enables the efficient expansion of CAR-T cells but also, through genetic engineering, endows them with the ability to secrete anti-PD-1 antibodies, thereby allowing them to actively counteract local immunosuppression upon reinfusion (98). Loading collagenase nanogels onto the surface of CAR-T cells to form a “backpack” enables the continuous degradation of collagen barriers ahead during cell migration, while simultaneously blocking misleading chemotactic signals. This significantly improves the infiltration and distribution of CAR-T cells within solid tumors (99). Utilizing acid-responsive nanoformulations to specifically release hyaluronidase at the tumor site for ECM degradation allows for the programmed remodeling of the matrix, thereby markedly enhancing the deep infiltration and killing capacity of adoptive NK cells (71).

#### 4.4.3 Multimodal synergy targeting mechanotransduction pathways

Combination strategies are evolving toward more complex cross-pathway interventions. Building on the analysis of the “stiffness-YAP-metabolism” axis, the combined use of YAP inhibitors with a low-leucine diet has been shown to synergistically suppress tumors in animal models by selectively inhibiting Tregs through the induction of mitochondrial dysfunction (19). Furthermore, novel strategies are emerging that aim to actively harness beneficial mechanical signals. For instance, designing personalized cancer vaccines with high stiffness can significantly enhance anti-tumor immune responses by activating the mechanosensitive channel Piezo1 in dendritic cells (100), thereby opening a new avenue for immunotherapy.

In summary, combining tumor matrix softening with immunotherapy has become a key direction for overcoming immune resistance in solid tumors. Future research should focus on advancing the clinical translation of these synergistic strategies and further elucidating the deep, interconnected regulatory networks between mechanical signals and processes such as immunometabolism and cell differentiation. This will lay the foundation for developing the next generation of combination therapies.

## 5 Discussion and prospects

Tumor matrix stiffness drives immunosuppression by directly affecting immune cells and reshaping the microenvironment. A key clinical question is whether these mechanical signals cause resistance to immunotherapy. Large-scale melanoma analysis confirms this: high expression of the mechanosensitive hub YAP1 predicts poor response to anti-PD-1 therapy and prognosis, directly guiding treatment (101). This links mechanical signals to immunotherapy resistance and establishes YAP1 as a critical resistance driver. Inhibiting YAP1 can reverse resistance and remodel the immune microenvironment (101), demonstrating that targeting aberrant mechanical pathways like YAP is a viable strategy to overcome ICI resistance. This also justifies intervening upstream via integrins or Piezo1. Breast cancer studies further show “immune-cold” tumors overexpress ECM stiffening genes (102), confirming stiffness hinders T cell infiltration and function (103). Together, this supports targeting the matrix to reverse immunosuppression.

The clinical relevance of targeting mechanosignaling is exemplified by recent advances with FAK inhibitors. The FAK inhibitor defactinib, in combination with the RAF-MEK inhibitor avutometinib, received accelerated approval from the FDA in May 2025 for recurrent KRAS-mutant low-grade serous ovarian cancer. This approval was based on a phase II trial (RAMP 201) demonstrating a confirmed objective response rate of 31% overall and 44% in KRAS-mutant patients, with a median duration of response of 31.1 months (104). Notably, only 10% of patients discontinued treatment due to adverse events, indicating a manageable safety profile. However, clinical progress of LOXL2-targeting agents has been limited. The LOXL2 antibody simtuzumab failed to demonstrate clinical benefit in phase II trials for pancreatic cancer and KRAS-mutant colorectal cancer (105, 106), highlighting the challenges in translating LOXL2 inhibition into clinical benefit. In parallel, preclinical evidence further supports the therapeutic potential of pirfenidone in modulating the tumor microenvironment. In an orthotopic pancreatic cancer mouse model, a nanodrug co-delivering gabapentin and pirfenidone remodeled CAF phenotypes, promoted inflammatory CAF accumulation, and enhanced functional CD8^+^ T cell infiltration, leading to a nearly two-fold increase in survival (85).

Translating these mechano-targeting strategies into therapies requires addressing their complexity. Mechanical signal regulation is context-dependent: often inhibitory in the TME, yet essential for immune activation in specific settings. For instance, Piezo1 promotes anti-tumor Th9 differentiation in CD4^+^ T cells (107) and pro-inflammatory Th1 responses in DCs (108). In NSCLC liver metastasis, agonizing Piezo1 synergizes with stiffness to create an ICI-sensitive microenvironment (109). Thus, non-selective blockade may harm beneficial immune signals. Future therapies must move beyond simple matrix softening to achieve precise, context-aware modulation.

Current single-target softening strategies are limited. Advanced research reveals crosstalk between stiffness, metabolism, and immunity, offering new reprogramming avenues. Activated CAFs rely on intense glucose metabolism to secrete ECM and sustain immunosuppression; disrupting their metabolism prevents stiffening and improves T cell infiltration (110). Stiffness, via YAP, can reprogram immune cell metabolism—e.g., steering Tregs toward OXPHOS to enhance suppression (17, 19). Combining mechanical pathway inhibitors with metabolism-disrupting agents may more effectively remodel the immune microenvironment.

A clinical translation pathway is emerging. First, precise patient stratification is essential, requiring non-invasive tools (e.g., ultrasound shear wave elastography (87)) to assess tumor stiffness and correlate it with the immune molecular landscape, identifying patients with “mechanical barrier”-driven resistance. Second, therapies must become intelligent and synergistic. For example, TME-responsive nanocarriers can deliver YAP inhibitors like verteporfin specifically to tumors (111). More complex systems, such as nanoparticles co-loading verteporfin and FSP1 siRNA, achieve triple synergy via YAP inhibition, photodynamic killing, and ferroptosis to activate immunity (112). Future combinations must also preempt side effects. An innovative approach co-assembled verteporfin with the mTOR inhibitor Torin 1, simultaneously killing tumors and blocking therapy-induced angiogenic/fibrotic signals, eliciting potent anti-tumor immunity (113). This marks the shift from single targets to reshaping the entire microenvironmental ecosystem.

In summary, targeting tumor matrix stiffness has matured into a translational field. Progress depends on precise, intelligent strategies, requiring deeper insight into mechanical signal duality and integration of mechanobiology, immunometabolism, materials science, and clinical medicine. Through interdisciplinary innovation, we can overcome barriers in solid tumor immunotherapy, reverse “cold” microenvironments, and offer transformative hope to patients.

## 6 Conclusion

**Figure 3:**
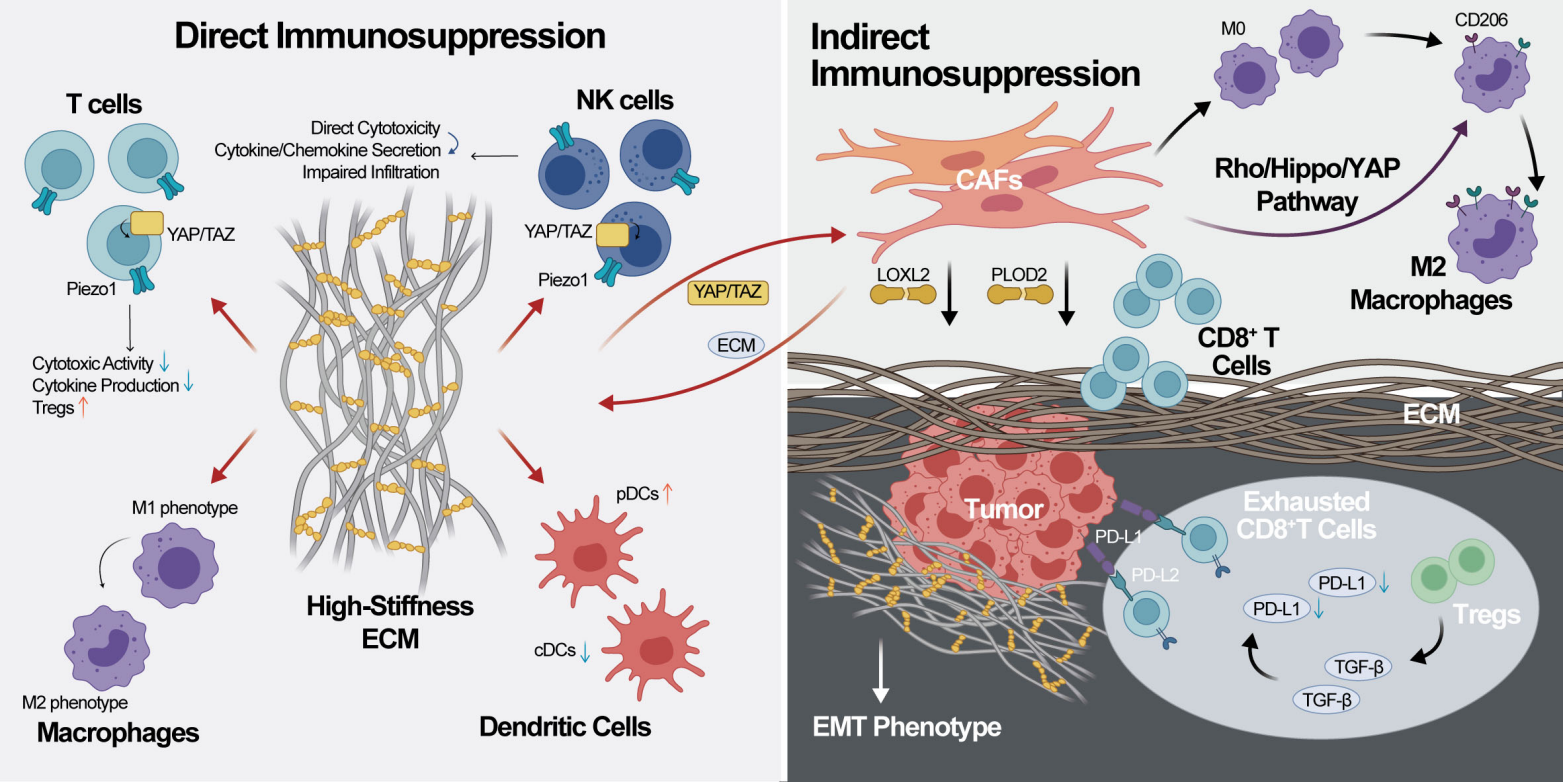
Graphical abstract summarizing the dual mechanisms of tumor extracellular matrix stiffness-driven immunosuppression. High-stiffness ECM directly suppresses T cell and NK cell functions and promotes Treg differentiation, M2 macrophage polarization, and pDC expansion via YAP/TAZ and Piezo1 mechanotransduction pathways. Indirectly, stiffness activates cancer-associated fibroblasts to remodel ECM via LOXL2 and PLOD2, induces tumor cell epithelial-mesenchymal transition, and upregulates immune checkpoints such as PD-L1, collectively establishing an immunosuppressive tumor microenvironment.

Tumor matrix stiffness drives immunosuppression through dual mechanisms: directly suppressing effector immune cells while promoting immunosuppressive populations via YAP/TAZ and Piezo1 pathways, and indirectly remodeling the microenvironment through CAF activation and EMT induction. These mechanical signals establish a self-reinforcing fibroinflammatory ecosystem that fosters immunotherapy resistance, with YAP1 emerging as a clinical biomarker of poor response. Therapeutic strategies targeting matrix stiffness—including enzymatic degradation, mechanotransduction inhibition, and anti-fibrotic drug repurposing—show promise in converting “cold” tumors to “hot” phenotypes when combined with immunotherapies. Future advances require precision mechano-immunomodulation, integration with metabolic interventions, and non-invasive stiffness monitoring for patient stratification. Targeting the mechanical dimension of the tumor microenvironment offers a transformative approach to overcome immunotherapy resistance in solid tumors (**Figure 3**).

## Glossary

ARF: Actomyosin Retrograde Flow
BET: Bromodomain and Extra-Terminal domain
CAF: Cancer-Associated Fibroblast
CAR-T: Chimeric Antigen Receptor T-cell
CCL5: C-C Motif Chemokine Ligand 5
CCR7: C-C Chemokine Receptor 7
cDC: conventional Dendritic Cell
CD: Cluster of Differentiation
cGAMP: cyclic GMP-AMP
cGAS: cyclic GMP-AMP Synthase
COX-2: Cyclooxygenase-2
CRC: Colorectal Cancer
CSF-1: Colony-Stimulating Factor 1
CXCL5: C-X-C Motif Chemokine Ligand 5
CXCR2: C-X-C Chemokine Receptor 2
DAB2: Disabled Homolog 2
DC: Dendritic Cell
ECM: Extracellular Matrix
EGCG: Epigallocatechin Gallate
EMT: Epithelial-Mesenchymal Transition
ERK: Extracellular signal-Regulated Kinase
FAK: Focal Adhesion Kinase
FSP1: Ferroptosis Suppressor Protein 1
GM-CSF: Granulocyte-Macrophage Colony-Stimulating Factor
GRHL3: Grainyhead-like transcription factor 3
HDAC3: Histone Deacetylase 3
HIF-1α: Hypoxia-Inducible Factor 1-alpha
HSPG2: Heparan Sulfate Proteoglycan 2
ICI: Immune Checkpoint Inhibitor
IGF-1: Insulin-like Growth Factor 1
IL-2: Interleukin-2
Lars2: Leucyl-tRNA Synthetase 2
;LH2: Lysyl Hydroxylase 2
LOX: Lysyl Oxidase
LOXL2: Lysyl Oxidase-Like 2
M1: Classically Activated Macrophages
M2: Alternatively Activated Macrophages
MDSC: Myeloid-Derived Suppressor Cell
MEK: Mitogen-Activated Protein Kinase Kinase
MMP: Matrix Metalloproteinase
mTOR: mechanistic Target of Rapamycin
NF-κB: Nuclear Factor kappa-light-chain-enhancer of activated B cells
NK: Natural Killer cell
NSCLC: Non-Small Cell Lung Cancer
Osr2: Odd-skipped related transcription factor 2
OXPHOS: Oxidative Phosphorylation
pDC: plasmacytoid Dendritic Cell
PD-1: Programmed Cell Death Protein 1
PD-L1: Programmed Death-Ligand 1
PFD: Pirfenidone
PI3K: Phosphoinositide 3-Kinase
PLOD2: Procollagen-Lysine,2-Oxoglutarate 5-Dioxygenase 2
PMN-MDSC: Polymorphonuclear Myeloid-Derived Suppressor Cell
RhoA: Ras homolog family member A
RNF114: Ring Finger Protein 114
ROCK: Rho-associated protein kinase
SHP-1: Src Homology 2 domain-containing Phosphatase 1
SLC20A1: Solute Carrier Family 20 Member 1
STAT3: Signal Transducer and Activator of Transcription 3
STEAP3: STEAP family member 3
STING: Stimulator of Interferon Genes
TAM: Tumor-Associated Macrophage
TAZ: Transcriptional co-Activator with PDZ-binding motif
TCA: Tricarboxylic Acid cycle
TEAD: TEA domain transcription factor
TGF-β: Transforming Growth Factor-beta
TME: Tumor MicroenvironmentTreg: Regulatory T cell
USP8: Ubiquitin-Specific Protease 8
VEGFA: Vascular Endothelial Growth Factor A
YAP: Yes-Associated Protein
YAP1: Yes-Associated Protein 1
ZEB1: Zinc Finger E-Box Binding Homeobox 1
α-SMA: alpha-Smooth Muscle Actin
